# Casting Processes from the Standpoint of the Metallurgist*Read before the Los Angeles County Dental Society, January, 10, 1922. Reprinted in “Pacific Dental Gazette,” March, 1922.

**Published:** 1922-05

**Authors:** Mearle W. Wilkinson


### CASTING PROCESSES FROM THE STANDPOINT OF THE METALLURGIST *

BY MEARLE W. WILKINSON, EM., MS.

At the invitation of one of your members, I have prepared a paper to present to you this evening, in which I have attempted to discuss certain metallurgical or chemical actions or reactions that are involved in the process of casting inlays, sections of bridge work, partial plates, clasps, etc., making use of the more or less complex gold alloys which have been developed in comparatively late years to adequately meet the requirements of cast dental restorations. It has not been my intention to overstep the bounds of my profession which allow me to present the casting process from the standpoint of a metallurgist, neither has it been my aim to discuss any serious fault coherent to the metallurgical apparatus or technique employed in the casting methods of today. The production of accurate and dependable gold castings is not controlled wholly by metallurgical knowledge, but due consideration must be given the physical characteristics of investment compounds, the wax of which the pattern is made, the method employed in burning out the wax, etc. The dental profession in the past years has devoted a great deal of time to these considerations and they have been developed to the extent of very dependable and uniform results. It is my firm belief that one of the greatest problems to the unlimited success of the casting process is one that is purely metallurgical in nature. I refer to the method by which gold alloys containing oxidizable metals are put into liquid form for casting, in which operation there is now no uniformity.

* Read before the Los Angeles County Dental Society, January, 10, 1922. Reprinted in "Pacific Dental Gazette," March, 1922.

The most important property of metals or precious metal alloys for use in casting dental restorations is their ability when in a melted state, of being cast into molds, filling completely every portion even the smallest cross-section of the latter. This important property is dependent on several other properties, for example, the melting temperature and closely allied to it, the fluidity, the direct or indirect method of melting and resulting from this melting operation, the development, absorption and occlusion of gases by the metal.

By melting temperature is meant that temperature at which all of the constituent metals of an alloy are thoroughly liquid. The lower the melting temperature, the more readily the material to be cast can be converted into the liquid state and the more conveniently it can be cast. Coin gold, 90% gold and 10% copper, melting point 1735 F., is more easily liquified and cast than an alloy, 90% gold and 10% platinum, melting point 2085 F. There are dental operations, however, such as subsequent soldering and resoldering that necessitate the melting temperature of gold alloys used in dentistry, being sufficiently high to allow for the use of the highest carat gold solder and it is well to choose casting golds with this in mind. The melting temperature of gold alloys is directly influenced by the proportion by weight of constituent metals. Briefly, this influence of the alloying metals now used in dental casting golds, on pure gold, melting point 1945 F., is as follows:

Copper although higher fusing, 1980 F., lowers the melting point, 10% by weight lowering it as much as 210 F.

Silver is almost neutral in its effect, 10% showing no appreciable lowering of the melting point.

Platinum, melting point 3190 F., raises the melting point very fast, 10% producing this effect to the extent of 140 F.

Palladium, melting point 2820 F., although lower fusing than platinum, raises the melting point of pure gold much faster, 10% raising it 205 F.

Iridium is so high fusing that it can not be used in dental casting golds in that it will not stay mixed, tending to segregate in casting.

Zinc, of course, is the metal used in gold solders to lower their fusibility.

The next property which exerts an influence upon the casting capacity of gold alloys is fluidity. The degree of fluidity again is dependent upon the composition to a certain degree, but more especially on the degree of overheating above the melting temperature in melting. The more thinly fluid the gold is without reaching the point of superheating, the more readily and the more completely it will fill the details of the mold. New gold is always more thinly fluid than gold that has been previously melted for the reason that absorbed gases and oxidation cause it to be sluggish. This, however, can be overcome in part by the correct use of the proper fluxes.

Many of the difficulties encountered in the casting of precious metal alloys such as occlusion of gases, brittle or incomplete castings, can be laid to oxidation of the so-called base metals with which the gold is alloyed. The noble metals, gold, platinum, palladium, ordinarily speaking do not oxidize. Metals such as silver and copper, essential to the strength of a precious metal alloy, do oxidize under the influence of atmospheric air, the air and gas blow-pipe or the oxygen and gas blow-pipe when melted previous to casting. If these oxides are not removed, they will enter the casting as such and a brittle gold results, the cohesion between the molecules of metal being broken by intermingled molecules of metallic oxides. A substance which possesses the property of combining with or reducing these oxides, forming with them a fusible slag, thereby increasing the fluidity of the melted metal is called a flux.

An oxidizing flux is one whose property is essentially oxidizing, such a flux being saltpetre (potassium nitrate) and borax. Nothing could be better to use in melting pure gold or metals that are non-oxidizable, copper and other base metals being impurities are oxidized and these oxides dissolved in the borax, thus purifying the gold as it is melted. When a button of alloyed gold has been melted and remelted a good many times without the addition of new gold, it has become so contaminated with oxides and other impurities that it is necessary to melt it thoroughly with a large quantity of oxidizing flux before it is in proper shape to be cast. After this melting, however, it no longer has the physical properties that it formerly possessed, having lost a great deal of its strength due to the removal of copper by the use of an oxidizing flux. This is an important consideration when using buttons that have been revived in this way. Hence an oxidizing flux is used only as a means of cleaning very dirty buttons and its use is always followed by a melting on a carbon block with a reducing flux.

A reducing flux is one possessing the property of combining with oxygen forming a liquid slag. The carbon which it contains has a chemical affinity for oxygen which is greater than that of the oxidizable metals, therefore, metallic oxides that may be formed during the process of melting are reduced to virgin metal by being relieved of their combined oxygen, the same going into the slag. It is apparent then that theoretically, no metallic oxides enter the gold casting and that no copper nor other base metal is lost in the process of melting and fluxing. This is not entirely true, however, under the severity of a highly oxidizing flame impinging directly upon a partially exposed metallic surface, but the benefit derived is so marked as to make the proper and constant use of a reducing flux essential in the correct handling of alloyed golds. All alloyed gold buttons previous to melting to be cast, should be melted thoroughly on a carbon block almost to a white heat, using large amounts of reducing flux, so that the button almost swims in the flux. When the button has solidified and has reached a dull red, chill in water to remove adhering flux, boil in a weak hydrochloric acid solution to remove surface oxidation and neutralize the acid by dipping into a concentrated soda solution. The button is then ready for casting. Just previous to applying pressure, whether a centrifugal or pressure type of casting machine, sprinkle just a minute quantity of the reducing flux on the surface of the melted metal. This will relieve surface tension and allow the metal to flow freely. Remember too, that the cleaner the flux the more freely the metal will flow. Flux that has been melted and remelted in contact with bodies that it dissolves, finally becomes sticky and sluggish when melted and is then an excellent drawback to free flowing gold.

Probably the most troublesome property of a gold alloy which materially impairs its casting capacity is its property of developing, of absorbing and of holding gases while in a fluid state. This property gives rise to the well-known blisters that appear in gold plate, the so-called pitted surfaces of gold castings and the further so-called spitting of gold alloys during melting. This spitting has been attributed by many to the burning out of copper even though the temperature attained is much below the melting point of copper. Alloys of gold containing no copper exhibit this same property of spitting. Cast pure gold and cast gold alloys are not dense in their structure as seen under the microscope. This means that between the molecules of metal there are minute spaces which are filled with entrapped gas or air. By cold working of cast metals such as rolling, hammering or drawing, a wrought condition is induced, in which the molecules of metal are compressed on themselves, increasing their density and thus increasing their specific gravity. The minute spaces between the molecules are eliminated in part, producing greater cohesion between the molecules, thereby increasing the strength of the metal and forcing out a large proportion of the gas locked in the cast metal. Oftentimes, however, a few pockets of gas remain after rolling, which are compressed and upon heating or annealing the gas expands and blisters are formed on the surface of the metal. The nature of the gas contained in these pockets is more or less a matter for conjecture and is no doubt variable under the conditions surrounding the process of melting. Most metals have an inherent property of absorbing gases when heated and especially while in a fluid state. If this development of gas takes place shortly before solidification, the gases can no longer escape and the resulting casting is full of gas bubbles. If this development or absorption of gas takes place at the start of melting and the gas is not removed before casting, the resulting casting will show the characteristic pitted cross-section due to occluded gas. Since the volume of these gases increases materially with the temperature, this process is more plainly perceptible with refractory or high fusing metals and with the use of high temperature and fast melting devices. The man who uses the ordinary gas and air blow-pipe in melting his casting golds never has the trouble from pitted golds that the man who uses oxygen and gas has, provided he has sufficient heat to thoroughly melt his gold. It takes him longer to bring his gold to the proper degree of fluidity and hence more time is allowed for the removal of the gases before casting. You have all noted the spitting which takes place when you hurriedly melt an ingot of casting gold under the intense heat of the oxygen and gas blow-pipe. This spitting is due to small pockets of gas which are expelled from the gold and which have been absorbed at the start. When the gold first begins to melt and sluggishly takes a spherical shape, it has not yet reached a period of complete fluidity. The absorbed gas can not get out under these conditions. If the button of gold is chilled at this point and cut in two, it will be found very porous throughout depending, of course, on the method of melting. As the melting is continued and the point of complete fluidity is reached, the gas pockets are free to move about, join one another and upon reaching the surface of the metal where the tension is no longer great enough to contain them, they force themselves out. When casting golds show this spitting therefore, it is a necessary adjunct to as dense a casting as is possible of a cast metal, because all gas pockets must be eliminated before they are carried with the gold into the mold. A little flux in connection with melting will have a tendency to take up a great deal of this gas and prevent in a degree this property of "spitting." The development of dissolved gases in a metal may be prevented sometimes by adding to the metal a body which enters with the dissolved gas into a non-volatile combination which does not again disintegrate. This must be a substance which has a great chemical affinity for the nature of the dissolved gases. Copper we know has a great affinity for oxygen. Silver absorbs and dissolves large quantities of oxygen in melting, making it difficult to cast pure silver without blowholes. If we add copper to the silver the absorbed oxygen chemically should combine with the copper. This in turn gives us copper oxide and as a result we have a metallic casting containing molecules of intermingled non-metallic casting containing molecules of intermingled non-metallic copper oxide and hence a casting the molecular cohesion of which is broken and therefore what we would term a more or less brittle casting. This apparently is not desirable in dental casting golds. If, however, the metal could be melted in casting with a large quantity of dissolving and reducing flux, as it can be on a carbon block, these oxides of copper would be removed by solution in the flux.

It would seem from what has gone before, that due to the many difficulties to be met with in melting and casting dental gold alloys, that it would be almost impossible to produce or expect to produce, accurate and dependable gold castings, at least without some change in the nature of the alloys that are presented to the dental profession by metallurgical science or in the methods that are used by the profession in melting. There are those of you, I am sure, who have experienced, some bearing in mind the fact that porous castings are not always and seldom are, the result of overheating of the gold, will see to it that the gold is free from entrapped gases and perfectly fluid before casting. There are those of you who have never or seldom ever meet with these troubles and it is to be hoped that you do not change your casting technique in any detail. There are so many possible variations that uniformity, a sought-for and necessary adjunct to successful casting, is gained only through careful manipulation, endeavoring to have as nearly as possible, the same combination of gases in the mixture from your blow-pipe, the same position of the flame on the metal, the use of the same amount of reducing flux and as nearly the same casting temperature as is possible. In connection with casting temperature as an aid to securing uniformity, it is well to choose the various casting golds for inlays, ridgework, clasps, etc., of as near the same melting or casting temperature as possible.

Perhaps the greatest source of lack of uniformity in casting is in the melting device and at the same time it can be blamed for most of the discouraging results of casting. The principle of casting oxidizable metals where the melting is accomplished by a direct open flame impinging upon the surface of the metal, is wrong without doubt. To prepare alloys containing base metals such as copper a reducing atmosphere is absolutely necessary. The same is true of the melting of these alloys after they are prepared. How to obtain these conditions in the melting and casting of dental casting golds is a problem still unsolved and a problem which if solved, will aid materially in producing gold castings of proper degree of strength and elasticity, free from porosity and following in every detail the lines of the mold. Such castings are now being made, it is true, but not uniformly so.

Owing to the fact that certain of your members have recognized the subject-matter of this paper as of the utmost importance to all of you and the time being limited this evening, it has been deemed advisable to present the concluding section of this paper on dental metallurgy at a future date. At that time I shall endeavor to have prepared, material covering the chemistry and metallurgy of conditions as they exist in the mold at the time of entrance of the fluid gold, the influence of cold working, annealing, tempering and pickling on the completed gold casting and the physical characteristics of dental casting golds, such as hardness, elasticity, denseness, shrinkage and resistance to acid reaction and the relation of these to the metallurgical composition of the alloy.

